# Encoding Scratch and Scrape Features for Wear Modeling of Total Joint Replacements

**DOI:** 10.1155/2013/624267

**Published:** 2013-04-11

**Authors:** Karen M. Kruger, Nishant M. Tikekar, Anneliese D. Heiner, Thomas E. Baer, John J. Lannutti, John J. Callaghan, Thomas D. Brown

**Affiliations:** ^1^Orthopaedic Biomechanics Laboratory, Department of Orthopaedics and Rehabilitation, University of Iowa, 2181 Westlawn Building, Iowa City, IA 52242-1100, USA; ^2^Department of Biomedical Engineering, University of Iowa, Iowa City, IA 52242, USA; ^3^Department of Materials Science and Engineering, Ohio State University, Columbus, OH 43210, USA

## Abstract

Damage to hard bearing surfaces of total joint replacement components typically includes both thin discrete scratches and broader areas of more diffuse scraping. Traditional surface metrology parameters such as average roughness (*R*_a_) or peak asperity height (*R*_p_) are not well suited to quantifying those counterface damage features in a manner allowing their incorporation into models predictive of polyethylene wear. A diffused lighting technique, which had been previously developed to visualize these microscopic damage features on a global implant level, also allows damaged regions to be automatically segmented. These global-level segmentations in turn provide a basis for performing high-resolution optical profilometry (OP) areal scans, to quantify the microscopic-level damage features. Algorithms are here reported by means of which those imaged damage features can be encoded for input into finite element (FE) wear simulations. A series of retrieved clinically failed implant femoral heads analyzed in this manner exhibited a wide range of numbers and severity of damage features. Illustrative results from corresponding polyethylene wear computations are also presented.

## 1. Introduction

Contemporary total hip and total knee arthroplasty (THA, TKA) procedures have excellent success rates clinically. However, in a few percent of cases, aseptic loosening due to wear-induced osteolysis remains a major impediment to implant longevity. This is especially a concern for patients with polyethylene bearings whose hard-surface counterfaces have been damaged due to scratching by 3rd bodies [1], or due to scraping from untoward events such as impingement or dislocation [2]. While it is well appreciated that damage of hard-surface counterfaces leads to elevated polyethylene wear, it is also recognized that differences in numbers, locations, and/or severities of such damage features have very different consequences in terms of wear rate elevation [3]. Unfortunately, despite the substantial morbidity of premature implant failures from accelerated polyethylene wear caused by counterface damage, a direct dose/response relationship between hard-counterface damage and accelerated polyethylene wear has yet to be elucidated. Part of the reason for this knowledge gap is that established damage characterization techniques for hard bearing surfaces do not describe that damage in a manner appropriate for direct, deterministic quantification of polyethylene wear. 

Detection of hard-surface damage features has commonly relied upon gross-level visual inspection and optical microscopy [4]. Scanning electron microscopy (SEM) provides enhanced information on the morphology of these damage features at yet higher magnification [1], but again mainly in the form of pictorial information. Quantification of surface morphology has primarily been done by means of profilometry. The majority of such work has involved stylus instruments, where fluctuations in the vertical position are recorded as the stylus physically moves short distances horizontally across the surface of interest [4, 5]. Since stylus profilometry recordings provide height variation data only along individual sampling lines, they have limited utility for quantifying the morphology of entire surfaces. Most commonly, therefore, investigators using this technique have resorted to spot samplings of presumed representative regions. For example, Hall et al. [6] quantified damage of retrieval femoral heads on the basis of 20 line profiles (each 1.4 mm in length) per specimen, taken in what was judged to be each specimen's most heavily scratched region. Besides being limited to surface height samplings along individual traverse lines, the short sweep length capacities of most line profile instruments also have made it difficult to quantify heterogeneous topography within large areas of damage [5].

High resolution maps of surface morphology can be generated through several techniques. Scanning tunneling microscopy transduces surface topography by monitoring the tunneling current flowing between an extremely sharp conductive probe and the sample surface. Atomic force microscopy generates three-dimensional images by means of a probe attached to the tip of a cantilever moving across the surface, monitoring the minute forces of interaction between the sample surface and probe [7]. Both of these techniques are able to measure surface height changes of less than a nanometer. However, sizes of the scan areas are extremely small, typically only in the range of a few ten-thousandths of a square millimeter. This limitation, plus the slow scan times involved, make these techniques impractical for use in mapping whole implant surfaces. Ultrasonic microscopes have been developed to examine surface mechanical properties of surfaces and to detect surface cracks and texture. However, these instruments have in-plane resolutions only on the order of a few tens of *μ*m, and they again require long scan times [8]. 

More recently, areal measurements of surface morphology have been facilitated by optical profilometry (OP). This technique captures surface features at subnanometer vertical resolution, using light interferometry [9]. (Laser illumination has also been used for interferometry instruments, but the coherence of laser illumination produces surface noise that is approximately twice as high as that for conventional light illumination [10].) OP's high vertical resolution is well suited to quantifying microscopic damage present on retrieval implant surfaces, with relatively high speed and high accuracy [10]. OP has been validated against stylus profilometry [11] and in turn has served as a gold standard for evaluating other imaging techniques [12]. The maximum sampling region size for OP scans is on the order of a few square millimeters. While most previous applications of OP have therefore still resorted to judgment-based spot samplings [9], OP scanning of selected substantial fractions of entire joint surfaces—while certainly tedious—is nevertheless tractable.

The measurements that are output from profilometry scans most commonly have been standard surface roughness parameters such as average roughness (*R*_*a*_), peak asperity height (*R*_*p*_), and maximum asperity depth (*R*_*v*_). While traditional roughness parameters of this class are straightforward to evaluate and interpret, their suitability for quantifying wear-consequential surface damage is less than ideal. For example, *R*_*a*_ is unable to distinguish between large groups of fine scratches versus small numbers of severe scratches. Similarly, *R*_*p*_ fails to differentiate between a single asperity versus multiple asperities of similar height. Also, these traditional roughness measures have normally been reported as isotropic scalar variables. Including the predominant directionality of microscopic-level damage is an important consideration, however, because physical wear tests have shown that differing angles between scratch orientation and the direction of relative surface motion can produce order-of-magnitude differences in wear rate elevation [13]. Moreover, for scrapes, the directionality of the microscratches within a given macro-level scrape is not necessarily coincident with the scrape's macro-level directionality. For example, the microscratches in scrape damage generated at an edge-loaded femoral head region during a THA dislocation event tend to be substantially askew to the macrodirection of the scrape [14].

While the microtopography of the hard-surface counterface is clearly a major influence on the rate of polyethylene wear [15], direct quantification of the damage-versus-wear relationship presently lacks physical basis. Rather, most work in this area has been empirical, usually involving simplified articulations such as those in pin-on-plate experiments [16]. At least for individual scratches, the best-correlating parameter in such work has tended to be scratch lip height, as reflected in *R*_*p*_ (peak asperity height). Even empirically, however, it has been difficult to identify statistically significant wear-versus-roughness relationships for the complex articulations characteristic of *in situ* function of whole implants. For example, in a study of 35 retrieval implants [6], there was only marginal correlation (*r* = 0.374, *P* = 0.099) between clinical wear factors and *R*_*a*_, with corresponding *R*_*p*_ values having an even weaker relationship with wear (*r* = 0.211, *P* = 0.225). 

In order to move beyond empirical observations, and to help underpin physics-based models of damage versus wear, it is essential to quantify the severity and directionality of individual scratches and scrapes. Novel image-based computational techniques developed for that purpose are here reported. These computational techniques for surface damage registry are applied to a series of total hip femoral heads that had been surgically retrieved following implant clinical failure. Characteristic aspects of whole-surface damage severity are reported for these retrievals. Finally, results are presented from an illustrative finite element computation of polyethylene wear acceleration associated with the damage to a specific femoral head from this series. 

## 2. Materials and Methods

Retrieval femoral heads were first digitally photographed using a novel diffused-light illumination technique [17]. This involved positioning the implant component on an angular indexing stage, inside a translucent white tube which eliminated spurious reflections from ambient lighting and room surroundings. Globally registered 1.6 megabyte digital photos (4432 pixels/mm^2^) were then taken from the polar direction and at 30 degree increments circumferentially, so as to image the entire bearing surface. These images provided vivid visual rendition of all macroscopically apparent damage features on the entire implant bearing surface. (As noted below, images taken at this resolution have been shown to highlight scratches that are well below wear-consequential severities.) The associated (grayscale) intensity modulations provided a basis for the damage to be objectively registered for purposes of image analysis. The damage was of two principal types: scratches and scrapes. The distinction was that scratches were manifest as thin discrete individual darkened lines, whereas scrapes were manifest as broad swaths of diffuse darkening, within which individual scratch tracks could not be distinguished at the global image level. 

**Figure 1:**
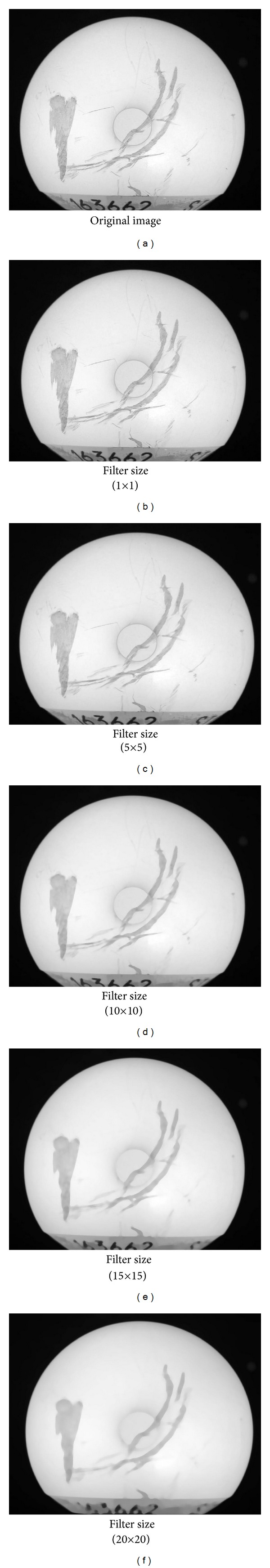
Successively stronger median filters of various sizes applied to an original image of a retrieval femoral head. The filter size indicates the neighborhood around the corresponding pixel in the input image for which the median value is calculated. This parameter allowed the analyst to control the distinction between scratch and scrape.

A custom-written MATLAB routine was used to determine regions of damage apparent in the global-level images. Canny edge detection was first performed to detect regions of damage, based on grayscale discontinuity relative to (bright) undamaged regions. This edge detector distinguished edges on the basis of maximum gradients of intensity. Edges were flagged if their gradients fell above an analyst-set threshold. This threshold value was set such that it was sensitive enough to detect the fine-scale scratches, but high enough so as to avoid influence from grayscale variations associated with the necessarily nonuniform distance from the camera lens. Next, a median filter was applied to the original images, replacing each pixel's intensity with the median of pixel intensities in a surrounding square region of analyst-specified size. Median filtering in this context had the effect of “blurring away” linear damage features (i.e., scratches) whose breadths were below a specific threshold. Analyst specification of the median filter size thus provided a basis for objectively distinguishing between scratches versus scrapes, for which different computational treatments were utilized for purposes of polyethylene wear modeling (see below). Empirically, various filter sizes were applied to representative original images that contained both scratches and scrapes (Figure 1). A 20 × 20 filter size was judged appropriate for distinguishing between scratches and scrapes, and was therefore used for *en masse* data processing in this retrieval series. 

**Figure 2:**
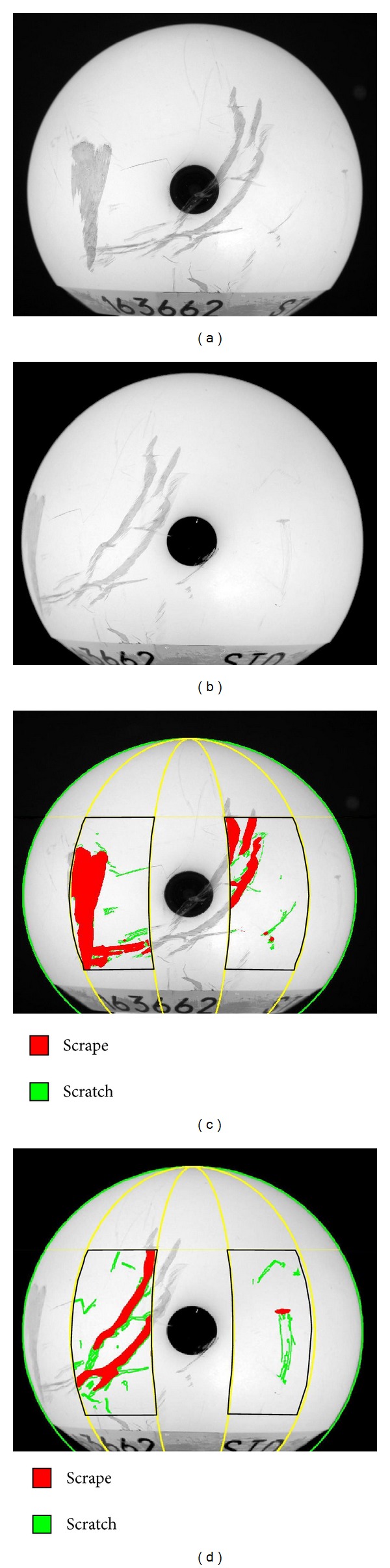
((a), (b)) Diffused-light images of a femoral head displaying both scratch and scrape damages, captured from two orientation directions 30° apart circumferentially. (The black dot in the center of the images is from camera lens reflection. This refection required that multiple view directions be utilized.) ((c), (d)) Image-processed results displaying identified damage regions. The sectors outlined in black indicate areas analyzed for this particular image. The top of the femoral head was captured and analyzed in companion polar images.

The darkened regions remaining after the median filtering operation were then autosegmented using an analyst-set intensity threshold. These constituted the scrape features. The scrape regions thus identified were then removed from the pre-median-filtered Canny edge detection result, leaving the remaining (i.e., non-scrape) damage features to be classified as scratches (Figure 2). A Hough transform was then used to discretize these scratch damage features into straight-line scratch segments. This analysis technique used a linear transform and the parametric equation of a straight line to determine the number of points falling along any given candidate straight line. Those candidate lines whose lengths fell above an analyst-set threshold were flagged, thus discretizing curvilinear scratches into concatenations of straight-line scratch segments. 

Next, OP was used to quantify the severity of each scratch or scrape. A Veeco Contour GT noncontact profiler (Bruker, Tuscon, AZ), which captured surface features by means of light interferometry, was employed for this purpose. The maximum resolution in a plane tangential to the target surface was 0.25 *μ*m^2^ per pixel, and the maximum out-of-plane resolution was 0.01 *μ*m. The instrument was capable of directly imaging individual areas of sizes up to ~1 mm × 1 mm, and it could autostitch those captured individual images into composite images sized up to ~5 mm × 5 mm. The instrument's internal software allowed for removal of the global (spherical) curvature that was present in the raw scans. The parameters of global spherical curvature were estimated using a least-squares-error algorithm to achieve a quadratic fit to the raw surface data. Removal of this spherical form from the raw data therefore enabled determination of local deviations from the native implant surface. The internal software also included capability to report standard surface roughness parameters (*R*_*a*_, *R*_*p*_, etc.). 

**Figure 3:**
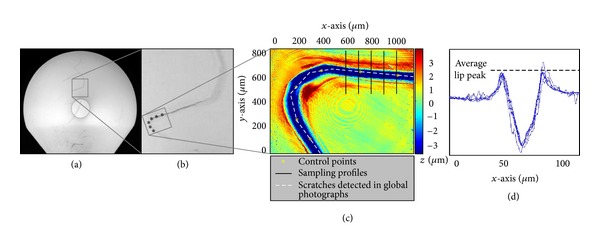
Global (a) and close-up (b) photographs of a scratched femoral head. Control points used for alignment with the OP scans are indicated by asterisks. (c) Local OP scan of selected region. (d) OP scan profiles used to calculate scratch lip height for a single scratch segment.

Superimposition of analyst-selected control points allowed alignment of the OP scans with their respective global-level images (Figures 3(a)–3(c)). For each OP scan, six sets of control points were chosen from damage features that were visually distinguishable on both the global-level image and the OP scan. These control points were used to compute a 2D transformation structure, based on second order polynomials. The global image was then translated and rotated based on this 2D transformation, to associate the detected damage features with their respective locations on the OP scan.

**Figure 4:**
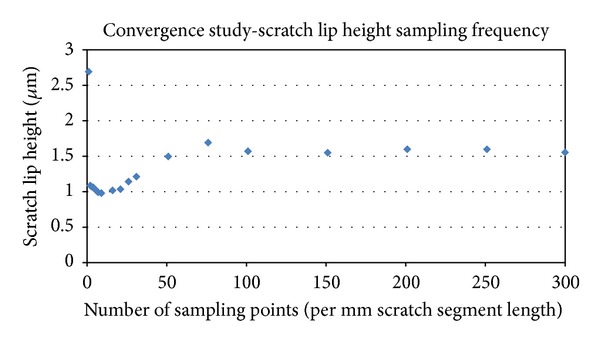
The results of a sampling convergence series, undertaken to determine appropriate sampling frequency.

Encoding the identified damage features involved accounting for both directionality and severity. In the case of scratches, directionality was simply the locally prevailing orientation of the scratch, and severity was based on the average scratch lip height (the dominant factor in wear elevation [18]) calculated from the OP data. To make the severity calculation, each designated scratch segment was first superimposed on its corresponding OP image(s) (Figure 3(b)). Values of surface vertical height were then queried from the OP dataset(s) along a series of equally spaced sampling lines directed perpendicular to the scratch segment. The peak surface height was identified along each of those sampling lines, and the mean of those peak heights was designated as the scratch lip height for that individual scratch segment (Figures 3(c) and 3(d)). (Convergence analysis had been performed to determine appropriate density of the sampling lines (Figure 4).) 

**Figure 5:**
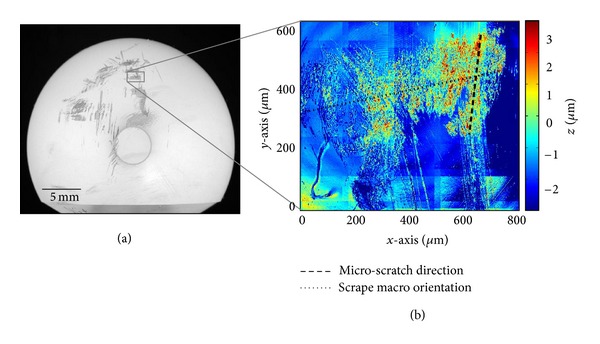
(a) Global photograph of scrape region. (b) Local OP scan, revealing scrape morphology. The dashed black line indicates the direction of microscratching, as determined by Hough transform. The dotted black line indicates the orientation of the macroscopic scrape.

In the case of scrapes, the directionality of interest for purposes of polyethylene wear modeling was not the macro-level direction of the scrape, but rather the direction of “microlevel” scratching within the scrape. While these two orientations were similar or nearly so for many scrapes, there were notable exceptions, such as when circumferentially oriented macroscrapes resulted from transverse sliding during egress of edge-loaded femoral heads during subluxation. To identify the direction of microscratching, a Hough transform was performed on each scrape OP dataset, which detected the microscratch lines within the scrape. The longest such line was designated as the scraping direction for that scrape area (Figure 5). Severity of scraping was quantified in terms of the average roughness within the segmented scrape region. This computation was made from OP data, again based on control point registration between (polygon-delineated) macrolevel scrape features and corresponding features in the OP datasets. The average roughness (*R*_*a*_) value within each scrape polygon was simply the average of the height differences of individual pixels, relative to the mean surface height of all the pixels within the scrape polygon. 

Current computational models of polyethylene wear in total joint replacements most commonly implement some form of the classic Archard wear formula [19]. This formula estimates local wear depth as the product of (1) contact pressure, (2) sliding distance, and (3) a wear coefficient dependent on the tribological characteristics of surface contact. For computing damage-induced wear rate acceleration, the Archard wear coefficient for the baseline undamaged surface is elevated for polyethylene areas that are overpassed by the counterface damage features. For scratches, there is an approximately exponential relationship [20] between scratch lip height (*h*_*L*_) and the scaling factor (*k*_inc_) for wear coefficient elevation, the specific parameters being

(1) $$\begin{array}{c} k_{\text{inc}}=58.0985-58.0985\cdot e^{-0.2237*h_{L}} \end{array}$$

when lip height is expressed in *μ*m. For the case of scrapes, the *R*_*a*_ values were converted to wear coefficient scaling factors using a power law relationship [16], the specific parameters being

(2) $$\begin{array}{c} k_{\text{inc}}=37.538\cdot {\left(R_{a}\right)}^{1.2} \end{array}$$

for *R*_*a*_ measurements in *μ*m.

## 3. Results

**Figure 6:**
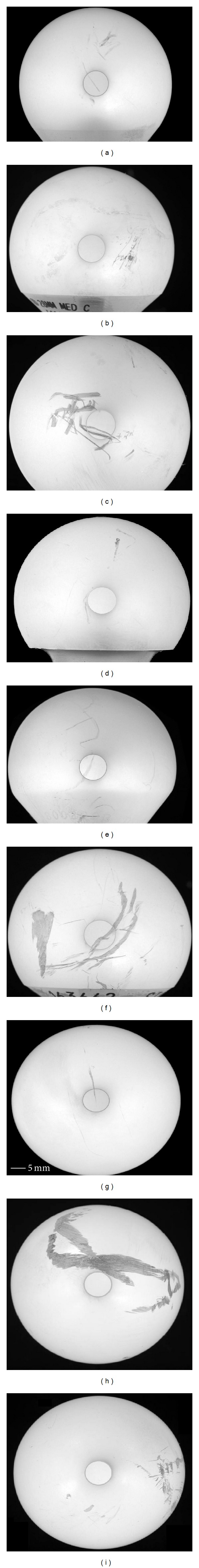
Global photographs of representative retrieval femoral heads.

**Figure 7:**
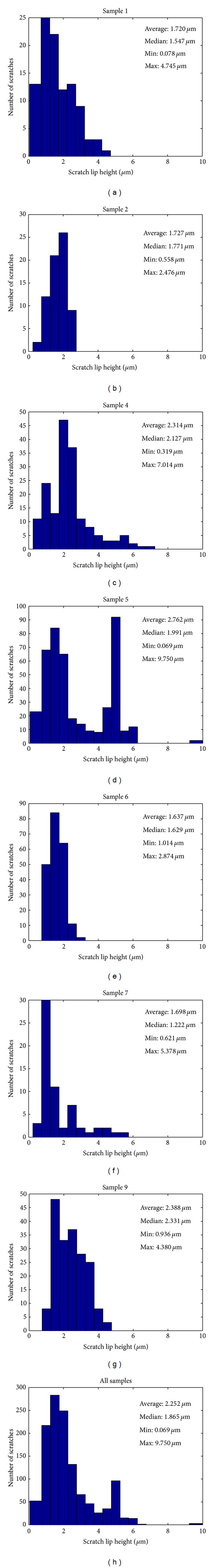
Distributions of the scratch lip height values for each femoral head that displayed scratch damage.

Scratch and scrape damage was encoded for nine representative specimens from our institution's collection of femoral head retrievals (Figure 6). The image processing routine detected scratches on seven of those specimens and scrapes on five of them. The distribution of severities of detected scratches (lip heights) and scrapes (*R*_*a*_ values) are reported in Tables 1 and 2, respectively. The seven specimens exhibiting scratch damage all showed large variability in the severity of individual scratches (Figure 7). The particular specimen showing the greatest amount of variability (specimen no. 5) had scratch lip heights ranging from 0.10 to 9.75 *μ*m. In the interest of completeness, the present set of results includes scratches with lip heights as low as 0.07 *μ*m. This may be unnecessarily exhaustive, however, since there is experimental evidence that the lip height threshold for detectable wear rate acceleration from individual scratches is substantially higher, on the order of 1 *μ*m [18]. If desired for purposes of computational economy, wear-inconsequential scratches can be disregarded from further consideration in downstream wear modeling, simply by Boolean masking on the basis of lip height.

**Figure 8:**
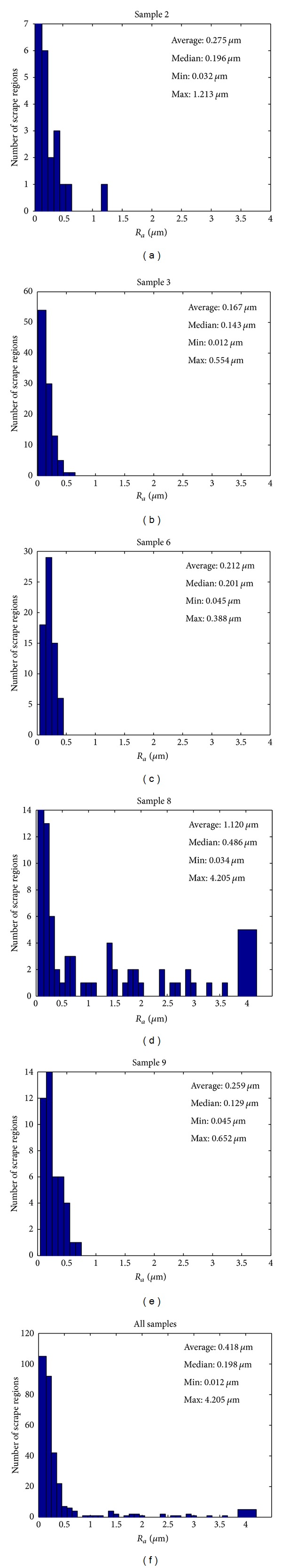
Distributions of the *R*_*a*_ values for each scrape region in the femoral heads that displayed scrape damage.

 The computed *R*_*a*_ values of scraped regions also showed considerable variability (Figure 8). Series-wide, the highest scrape-average roughness value was 4.20 *μ*m (specimen no. 8), a three-order-of-magnitude elevation relative to typical *R*_*a*_ values for undamaged implant surfaces [16].

These damage features can produce substantial increases in local wear rates. The presence of the most severe scratches increased the local wear coefficient by a factor of approximately 50, as shown by (1). The most severe scrapes regions produced wear coefficient increases of over 200-fold, as shown by (2).

## 4. Discussion

This collection of scratch hip heights and scrape *R*_*a*_ values, along with their associated individual directionalities, demonstrates a range of damage features on typical retrieval femoral heads. While the present OP data captures were similar to those for other OP applications [9, 21], the data from these computational techniques represent the first-global-implant level registry of micron-level damage features. Such datasets can be used as inputs to FE liner wear models, to allow clinically realistic simulation of femoral head damage on a case-specific basis. These data allow for damage representation on a scratch- or scrape-specific basis, and they allow for wear to be predicted due to each damage feature. This constitutes an improvement over previous methods, which have only represented damage in terms of standard surface roughness parameters and have been unable to establish strong linkage between any of these parameters and wear acceleration [6]. 

**Figure 9:**
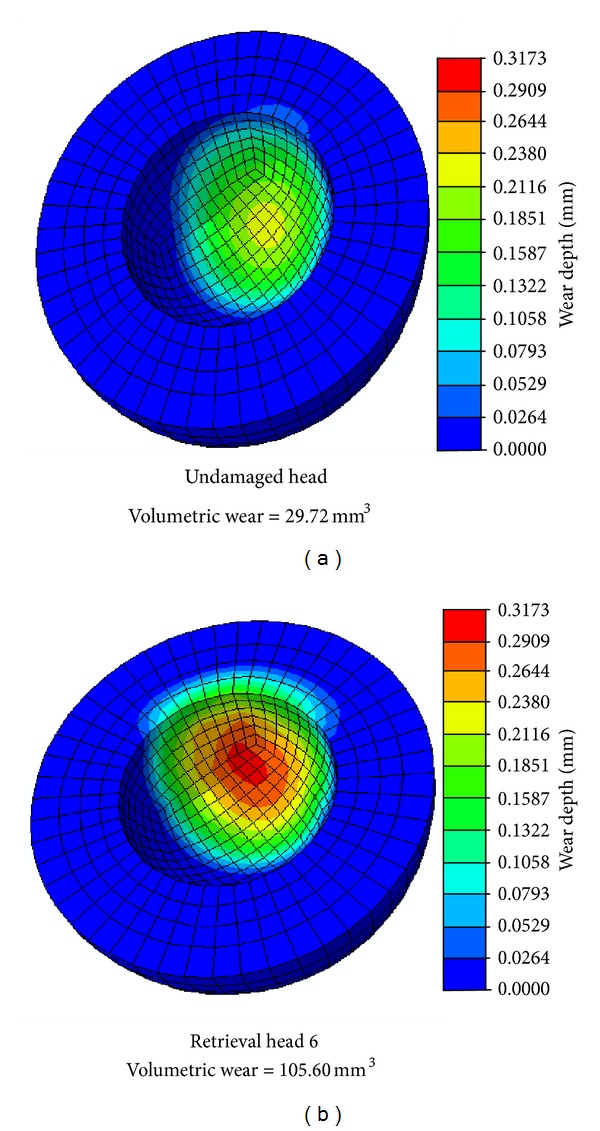
Computed wear depths (1 × 10^6^ cycles) for an undamaged and retrieval femoral head (28 mm).

Illustratively, an FE wear model of one particular specimen (specimen no. 6) from this series was generated by mapping each identified damage feature's severity, orientation, and global-level geometry onto the femoral head. Collectively, these damage features, comprised of a total of 211 scratch segments and 68 scrape regions, led to a 3.8-fold increase in polyethylene liner wear, compared to a baseline simulation of an otherwise-identical undamaged femoral head (Figure 9). Of this wear increase, 68% was due to the scratch damage and 32% was due to the scrape damage. 

Wear rate increases computed using this technique have been physically validated both for isotropically roughened patches [22] and for scratches [20]. While direct physical validation has not yet been performed for directional scrapes, the only difference between FE wear simulations for directional scrapes versus for isotropically roughened patches is the incorporation of directionality into the wear factor. 

The present series was restricted to total hip femoral component retrievals. This same damage registration framework presumably could also be used to characterize hard counterfaces in other total joint replacements such as the femoral components of total knees, which often exhibit damage features similar to those catalogued here. For example, a 2D interferometry study of TKA retrievals [23] identified both fine, closely packed scratches with lip heights on the order of 0.5 *μ*m, and severe damage features with scratch lips approaching 4 *μ*m. 

Quantification of femoral head damage through this analysis framework relies on the global diffused-light digital photographs and corresponding edge detection computations to identify damage features. While the diffused lighting technique in almost all instances provided much more vivid rendition of damage features than was apparent visually under room lighting, there were several instances where fine scratches that were visually apparent under normal room lighting were not visible in the diffused-light images. The OP scans of such “disappearing” scratches showed that these fine scratches had extremely low lips, indicative of their not being critical in terms of the end goal of determining wear rate acceleration. Further work to elucidate the relationship between diffused-light image grayscale values versus corresponding scratch lip heights may offer insight in this seemingly anomalous phenomenon.

## 5. Conclusion

For total joint replacements with a polyethylene bearing surface, dramatically accelerated wear is often associated with accrual of scratch or scrape damage to the hard-surface counterface. Moving from qualitative to quantitative assessments of this interaction requires a basis for representing hard-surface damage in a manner conducive to performance of physics-based wear analyses. The present paper reports a multiscale experimental/computational framework for making such damage representations. Global-level and microlevel imaging are coupled to computationally register the severity and directionality of both scratch and scrape damage present on entire implant bearing surfaces. This damage registry framework proved practical for use for typical retrieval total hip implants, thus opening the way for quantitative analyses of damage-related polyethylene wear rate acceleration on a case-specific basis.

To the authors' knowledge, the present datasets constitute the first-ever compilations of whole-surface damage features on orthopaedic total joint replacements. Besides their usage in the context of enabling wear computations, such datasets will likely prove useful in other contexts, such as for forensic assessment of specific surface damage events, and “reverse engineering” of implant designs to minimize wear-consequential counterface damage. 

## Figures and Tables

**Table 1:** Scratch lip heights on samples displaying scratch damage.

Femoral head	Number of scratch segments	Average scratch segment length (mm)	Scratch lip height (*μ*m)		
			RowSpanEmpty	RowSpanEmpty	Mean ± SD	Min	Max
1	101	0.59	1.72 ± 0.95	0.08	4.75
2	71	0.41	1.73 ± 0.46	0.56	2.48
4	172	0.91	2.31 ± 1.29	0.32	7.01
5	430	0.58	2.76 ± 1.73	0.07	9.75
6	211	1.03	1.64 ± 0.40	1.01	2.87
7	62	0.76	1.70 ± 1.13	0.62	5.38
9	190	0.87	2.39 ± 0.81	0.94	4.38

**Table 2:** Comparison of average roughness (*R*_*a*_) values on samples displaying scrape damage.

Femoral head	Number of scrape regions	Average scrape area (mm^2^)	*R*_*a*_ (*μ*m)		
			RowSpanEmpty	RowSpanEmpty	Mean ± SD	Min	Max
2	22	1.57	0.28 ± 0.27	0.03	1.21
3	104	1.20	0.17 ± 0.11	0.01	0.55
6	68	5.09	0.21 ± 0.08	0.05	0.39
8	71	8.03	1.12 ± 1.25	0.03	4.20
9	44	1.05	0.26 ± 0.15	0.05	0.65
